# Medicine donations: a review of policies and practices

**DOI:** 10.1186/s12992-023-00964-3

**Published:** 2023-09-01

**Authors:** Hannah Permaul Flores, Jillian C. Kohler, Deirdre Dimancesco, Anna Wong, Joel Lexchin

**Affiliations:** 1 WHO Collaborating Centre for Governance, Accountability and Transparency in the Pharmaceutical Sector, University of Toronto, Toronto, Canada; 2 Faculty of Arts and Sciences, University of Toronto, Toronto, Canada; 3 Leslie Dan Faculty of Pharmacy, University of Toronto, Toronto, Canada; 4 Dalla Lana School of Public Health, University of Toronto, Toronto, Canada; 5 Munk School of Global Affairs and Public Policy, University of Toronto, Toronto, Canada; 6 Department of Health Products Policy and Standards, World Health Organization, Geneva, Switzerland; 7 University of Toronto Faculty of Law, Toronto, Canada; 8 Temerty Faculty of Medicine, University of Toronto, Toronto, Canada; 9 Department of Health, York University, Toronto, Canada

**Keywords:** World Health Organization, Pharmaceutical industry, Non-governmental organizations, Pharmaceutical donation policies, Global humanitarian aid, Health policy

## Abstract

**Background:**

To help promote the effective delivery of drug donations, the World Health Organization (WHO) developed the *Guidelines for Medicine Donations*. The need for revisions is timely given the large-scale influx of medicine donations since the start of the COVID-19 pandemic. This study analyses current policies of donors and recipients that are commensurate with the recommendations in the Guidelines and examines current practices, challenges, and revision suggestions.

**Results:**

A search for medicine donation policies of donors and recipients was conducted in May/June 2022 and repeated in January 2023. Potential donor countries were identified from the high-income countries on the United Nation’s (UN) List of G20 Countries. Potential pharmaceutical company donors were selected from those with 2021 revenue of $30 billion or greater. Potential non-government organization donors came from the WHO list of non-governmental organizations (NGOs) and two other sources. Potential recipient countries were those on the UN List of Least Developed Countries. These four lists were supplemented with actual donors and recipients identified from the literature. All policies retrieved were screened to identify which of the 12 recommendations from the WHO *Guidelines* were incorporated. We identified 38 policies from 1 donor country, 6 brand-name multinational pharmaceutical companies, 6 NGOs and 25 recipient countries. Most policies incorporated all 12 recommendations. Twenty-five of the 38 policies were developed in 2010 or later. The majority of actual donors and recipients did not have policies that were publicly available. A rapid literature review for publications from 2010 onwards identified challenges in implementing the WHO *Guidelines* and suggested for revisions. Challenges included: (1) information management; (2) medication presentation; (3) influence from the pharmaceutical industry; (4) donation sustainability; and (5) the belief that donations are inherently good.

**Conclusions:**

Our findings suggest that both donors and recipients could further align their policies with the existing *Guidelines* and both groups should be consulted on any revisions to ensure that their experiences are reflected and their needs are addressed. While the current WHO Guidelines for Medicine Donations are a solid base for medical humanitarian efforts, evidence points to the need for an update to meet current challenges.

**Supplementary Information:**

The online version contains supplementary material available at 10.1186/s12992-023-00964-3.

## Background

Medicine donations ideally are beneficial insofar as they can enhance a population’s access to pharmaceutical supplies, saving lives and supporting health systems [1]. However, if not well coordinated or managed, medicine donations may impose unintended but costly administrative and resource allocation burdens on recipient countries [2]. What is more, medicine donations are often not done according to best practices and sometimes can create more burden than benefits for the governments they are intending to help [3]. To facilitate the need for donations to follow core principles and transparent best practices for medicine donations, the first edition of the World Health Organization (WHO) *Guidelines for Drug Donations* was published in May 1996 [4]. Developed in collaboration with United Nations (UN) agencies, international humanitarian relief agencies, and other non-governmental organizations operating in the development assistance space, the guidelines provided countries with a series of standardized recommendations for the development of national and institutional medicine donation policies.

A revised second edition, *Guidelines for Drug Donations* was published in 1999 following a global review of country experiences using the first edition [5]. The review underscored the need for a number of revisions to the *Guidelines* including greater flexibility to promote enhanced adherence, without which medicine losses were more likely to occur. Accordingly, the third and latest edition, *Guidelines for Medicine Donations*, was published in 2010 and builds on lessons learned from the implementation of the previous editions [6]. The *Guidelines* contain twelve best practice recommendations for donor and recipient countries. These are:

1. All medicine donations should be based on an expressed need, should be relevant to the disease pattern in the recipient country, and quantities should be agreed between donor and recipient.
2. All donated medicines or their generic equivalents should be approved for use in the recipient country and should appear on the national list of essential medicines or equivalent or in the national standard treatment guidelines, if the NEML [National Essential Medicines List] is not updated. Or, if a national list is not available, it should appear on the WHO model lists of essential medicines, unless specifically requested otherwise and provided with a justification by the recipient.
3. The presentation, strength, and formulation of donated medicines should, as far as possible, be similar to those of medicines commonly used in the recipient country.
4. All donated medicines should be obtained from a quality-ensured source and should comply with quality standards in both donor and recipient countries. The WHO Certification Scheme on the Quality of Pharmaceutical Products Moving in International Commerce should be used.
5. No medicines should be donated that have been issued to patients and then returned to a pharmacy or elsewhere, or that have been given to health professionals as free samples.
6. After arrival in the recipient country all donated medicines should have a remaining shelf-life of at least one year. Large quantities of donated medicines become a logistical challenge, even with a long shelf-life. Therefore, based on the national consumption and available quantities in stock or in the supply chain pipeline, all donated quantities should match the needs to be consumed before they are expired.
7. All medicines should be labelled in a language that is easily understood by health professionals in the recipient country. The label on each container should contain at least the International Nonproprietary Name (INN) or generic name, batch number, dosage form, strength, name of manufacturer, country of manufacture, quantity in the container, storage conditions and expiry date.
8. Donated medicines should be presented in pack sizes that are suitable for the recipient and appropriate to the setting in which they will be distributed or dispensed.
9. All medicine donations should be packed in accordance with international shipping requirements and should be accompanied by a detailed packing list that specifies the contents. The weight per carton should preferably not exceed 30 kg. Shipments of medicines should not be mixed with other supplies, unless they are shipped as kits with predetermined contents.
10. Medicine donations should be jointly planned, and collaboration between donors and recipients should begin early. Medicines should not be sent without prior consent of the recipient.
11. In the recipient country the declared value of a medicine donation should be based on the wholesale price of its generic equivalent in the recipient country, or, if such information is not available, on the wholesale world-market price for its generic equivalent.
12. Costs of international and local transport, warehousing, port clearance and (customs) storage, handling and disposal or reverse logistics of expired donated products should be paid for by the donor agency, unless specifically agreed otherwise with the recipient in advance.

Four core principles underly these practices. These are:

- Medicine donations should benefit the recipient to the maximum extent possible. As a result, all donations should be based on an expressed need and unsolicited donations are discouraged.
- Medicine donations should be made in compliance with the recipient country’s prevailing government policies and administrative arrangements.
- Effective coordination and collaboration should be fostered between medicines donor and recipient countries, with all donations made according to a plan formulated by both parties.
- No double standard in product quality should be considered acceptable. If the quality of a product is unacceptable to a donor country, it is unacceptable for that product to be donated to a recipient country.

Evidence-based reviews of country compliance with the 2010 *Guidelines*, undertaken in 2015 [7] and 2019 [8] respectively, recommended that WHO implement further revisions to the *Guidelines*. Cañigueral-Vila et al. [7] discussed the need for specific wording, inclusion of donation checklists and contrasting between acute and long-term emergency situations. McDonald et al. [8] concluded that most of the 53 donations that they identified “did not comply with at least some items in WHO guidelines and no reports provided sufficient information to assess compliance against all items.” They suggested there be an emphasis on encouraging donors and recipients to regularly report the status and completion of medicine donations.

Building on those reviews, this paper analyses current medicine donation policies developed by donor and recipient countries and key organizations. It evaluates congruence between their policies and the *Guidelines* recommendations and proposes recommendations for further revisions based on a survey of the literature focused on guideline implementation challenges.

## Methodology

### Identification of possible donors and recipients

We identified and screened three groups of possible donors and one group of possible recipients. We refer to “potential” donors and recipients because in advance of our search we did not have any knowledge about whether these actors had actually participated in donations. The definition of a donor country was when the government of a country arranged a donation. In contrast to the definition of a donor country, a recipient country was defined as the country where the donation was received regardless of whether the actual recipient was the government or an institution/organization within the country.

Potential donor countries were identified in a two-step process. We initially used the G20 list of countries because they collectively represent 84% of the global economy, [9] and are financially capable of providing large donations of medicines as part of their foreign or humanitarian aid programs. We then selected the high-income members of the G20 based on their identification by the World Bank (https://datahelpdesk.worldbank.org/knowledgebase/articles/906519-world-bank-country-and-lending-groupsSa).

Potential pharmaceutical company and non-governmental organization (NGO) donors were identified based on revenue and global footprint. Pharmaceutical companies examined were those with 2021 revenues larger than $30 billion. NGOs included for examination were those listed on a combination of Human Rights Careers’ list of “Biggest NGOs in the World” (https://www.humanrightscareers.com/issues/biggest-ngos-in-the-world/), the WHO list of Non-governmental Organizations (https://www.who.int/emergencies/partners/non-governmental-organizations) and suggestions from one of the authors (DD) who works in the WHO Department of Health Products Policy and Standards.

Potential recipient countries were identified based on United Nation’s Least Developed Countries (LDCs) status, with no restriction on geographic scope (https://unctad.org/topic/least-developed-countries/list). Recipient countries were limited to LDCs because these countries are those most likely to face significant financial barriers to access, and thus the most likely candidates for medicine donations (particular during times of public health crisis). In addition, countries in the LDC group were the most likely to be listed as donation recipients in the article by McDonald et al. [8].

Finally, the list of examined donors and recipients was cross-referenced against the list of actual donors and recipients named in the article by McDonald et al., as it is the most comprehensive and recent list of drug donations. Donors and recipients identified within that article were added to the list of potential donors and recipients if they had not yet been identified. The income status of these additional recipient countries was determined in reference to their World Bank income classification (https://datahelpdesk.worldbank.org/knowledgebase/articles/906519-world-bank-country-and-lending-groups).

### Identification of drug donation policies among possible and actual donors and recipients

Searches for policies were conducted in May and June 2022 and then repeated between January 20–26, 2023. A Google search of the websites of each donor and recipient country was undertaken using the terms “[country name]” and “policy on drug donations”. A Google search of the website of each potential pharmaceutical company and NGO donor was undertaken using the terms “product donations” and “policies”. All policies were screened by two members of the research team, with disagreements resolved by consensus. The source of the policy, actor identity, name of the policy, year it was issued and the URL link to the policy were recorded. Policies were analyzed to evaluate implementation of all 12 WHO *Guidelines*. Only policies in English were analyzed.

### Identification and analysis of the literature on WHO guideline implementation challenges and recommended revisions

A rapid literature review of the academic and grey literature on medicine donation policies was conducted in May and June of 2022 using the following databases: MEDLINE, PubMed, the Cochrane Library, and ScienceDirect. Rapid reviews are a form of evidence synthesis that can provide more timely information compared with standard systematic reviews and are typically done in less than 5 weeks. The search terms employed were: “disaster relief medicine,” “medicine donation policy,” and “pharmaceutical company donations.” Documents were analyzed to identify the difficulties in implementing donations and recommendations for revision of the WHO *Guidelines.* Only documents written in English were used. Documents exclusively dealing with medical device donations were excluded.

### Data analysis

The number of actors in each donor or recipient category with and without policies on donations was recorded. This was then cross-referenced with group of actors with both documented participation in past donations and who maintained active drug donation policies. Only descriptive data are reported.

## Results

We searched for donation policies among potential and actual donors and recipients from 12 donor countries, 24 pharmaceutical companies (22 multinational and 2 generic companies), 41 NGOs and 66 recipient countries (46 LCDs, 11 lower-middle countries, 8 upper-middle income countries and 1 high-income country). Table 1 provides information about these actors, their donor or recipient status, the date when their donation policies were developed, the WHO recommendations that they incorporate into their policies, and URL links to those policies. A full list of the surveyed donors and recipients without publicly available policies or guidelines on drug donations is available in Supplementary File 1.

Drug donation policies were identified for 1 donor country, 6 brand-name multinational pharmaceutical companies, 6 NGOs and 25 recipient countries (16 LCDs, 6 lower-middle income and 3 upper-middle income countries). (In addition, the United States Food and Drug Administration provided a set of questions and answers for public donations – see: https://www.fda.gov/media/80536/download.) Twenty-five of the 38 policies were developed in 2010 or later, i.e., at the time or after of the latest revision of the WHO *Guidelines*, while 7 policies were undated. Out of those 25 recently developed policies, 16 had all 12 WHO recommendations.

Australia was the only donor country found to have a national publicly available policy, incorporating 11 of the 12 WHO recommendations and missing recommendation 11: the declared value of a medicine donation should be calculated based on the wholesale price of its generic equivalent. All pharmaceutical donors implemented all 12 of the WHO recommendations. Only 4 of the 6 NGO donors implemented all 12 of the WHO recommendations, with the remaining two only implementing 3 recommendations – 1 (all donations should be based on expressed need), 4 (all donated medicines should be obtained from a quality-ensured source) and 12 (all costs associated with the donation should be paid by the donor). Thirteen of the 25 recipient countries implemented all 12 WHO recommendations. The recommendations most often excluded from recipient countries’ drug donation policies were recommendations 6 (donated medicines should have a remaining shelf-life of at least one year), 9 (medicine donations should be packed according to international shipping requirements, accompanied by a detailed packing list, not exceed 30 kg and not be mixed with other supplies) and 11 (the declared value of a medicine donation should be calculated based on the wholesale price of its generic equivalent).

Of the 5 donor countries, 20 donor pharmaceutical companies, 15 donor NGOs, and 26 recipient countries specifically mentioned in the McDonald et al. article, only 1 donor country, 3 donor pharmaceutical companies, 3 donor NGOs and 13 donor recipient parties had identifiable drug donation policies in place (Table 2). Supplementary File 1 provides further details about which of the donors and recipients examined in this study and the McDonald et al. article lacked drug donation policies.

**Table 1:** Donor and recipient actor policy alignment with the 2010 WHO *Guidelines for Medicine Donations*

Actor Identity	Donor or Recipient	Documented participation in donation activities*	Policy Name	Year of Policy	Incorporated WHO *Guidelines* Recommendations	Policy URL
Australia	Donor country	No	Australian Guidelines for Drug Donations to Developing Countries	2001	1–10, 12	http://returnmed.com.au/wp-content/uploads/2017/08/Australian-Guideline-re-drug-donations.pdf
AbbVie	Brand-name multinational pharmaceutical company donor	No	Our Commitment to Product Donations	No date	All 12	https://www.abbvie.com/content/dam/abbvie-dotcom/uploads/PDFs/our-commitment-to-product-donations.pdf
AstraZeneca	Brand-name multinational pharmaceutical company donor	No	Securing Our Future: Our Sustainability Journey	2016	All 12	https://www.astrazeneca.com/content/dam/az/Sustainability/55229%20AZ_Our%20Sustainability%20Journey_AW6_V2.pdf
Merck	Brand-name multinational pharmaceutical company donor	Yes	Charitable Product Donations	2019	All 12	https://www.merck.com/wp-content/uploads/sites/5/2020/04/Policy_2019_Charitable-Product-Donations_MERCK.pdf
Merck KGaA	Brand-name multinational pharmaceutical company donor	Yes	Charter on Access to Health (A2H) in Developing Countries: Pharmaceutical Product Donations	2016	All 12	https://www.emdgroup.com/company/responsibility/us/regulations-and-guidelines/A2H_Charter_infographic_ProductDonations.pdf
Novartis	Brand-name multinational pharmaceutical company donor	Yes	Donations	No date	All 12	https://www.novartis.com/esg/access/donations
Roche	Brand-name multinational pharmaceutical company donor	No	Policy on Philanthropic Donations and Non-commercial Sponsorship	2018	All 12	https://assets.cwp.roche.com/f/126832/x/0cbad5bee8/policy_on_donations_and_non-commercial_sponsorship.pdf
Catholic Relief Services	NGO donor	No	Supply Chain Management: Standard Operating Procedures	2011	All 12	https://www.crs.org/sites/default/files/tools-research/supply-chain-management-standard-operating-procedures.pdf
Direct Relief	NGO donor	Yes	Prescription Drug and Device Regulatory Compliance Policy	No date	All 12	https://www.directrelief.org/wp-content/uploads/prescription_drug_and_device_regulatory_compliance_policy.pdf
International Health Partners/ Medshare International	NGO donor	Yes	Donation Platform	2019	1, 4, 12	https://www.ihpuk.org/donation-platform
InterAction	NGO donor	No	InterAction NGO Standards	2018	All 12	https://www.interaction.org/documents/interaction-ngo-standards/
Medecins sans Frontieres	NGO donor	No	MSF Policy for In-Kind Donations of Medical Products and Health Technology	2018	All 12	https://www.msf.org/sites/default/files/msf-policy-in-kind-donations-medical-products-health-technology-2018.pdf
Project HOPE	NGO donor	Yes	Gifts in Kind: Everything You Need to Know	No date	1, 4, 12	https://www.projecthope.org/gifts-in-kind-everything-you-need-to-know/
Afghanistan	LDC recipient country	No	Afghanistan National Medicines Policy	2014	1–7, 9, 10	https://www.academia.edu/15138307/ Ministry_of_Public_Health_AFGHANISTAN_ NATIONAL_MEDICINES_POLICY
Bhutan	LDC recipient country	No	Bhutan Rules and Regulation	2019	1, 2, 6, 7, 12	https://bhutan.eregulations.org/media/Final%20BMR%202019.pdf
Bolivia	Lower-middle income recipient	Yes	National Drug Policy	1996	1, 2, 4, 5, 7, 9	https://www.global-regulation.com/translation/bolivia/3100449/national-drug-policy.html
Cuba	Upper-middle income recipient	Yes	Necessary Information for Import Permit Request: Appendix 1: Medicines	No date	1, 4, 5, 6, 7, 9, 11	https://www.canadiannetworkoncuba.ca/CNC/Donations_to_Cuba.html
Ethiopia	LDC recipient country	No	Ethiopian Food and Drug Authority: Guideline for COVID-19 Vaccine Donation	2021	All 12	https://covidlawlab.org/wp-content/uploads/2021/07/Guideline-for-COVID-19-Vaccine-donation.pdf
Fiji	Upper-middle income recipient	Yes	Guidelines for Donations of Medicines Medical Supplies and Equipment to The Republic of Fiji Islands	2016	All 12	https://www.health.gov.fj/wp-content/uploads/2018/03/Guidelines-for-Donations-of-Medicines-Medical-Supplies-and-Equipment.pdf
Gambia	LDC recipient country	No	Guidelines for Donation of Medicines and Related Products	2021	All 12	https://www.mca.gm/wp-content/uploads/2021/08/MCA-GL-010_v1_Donations_19May21.pdf
Ghana	Lower-middle income recipient	Yes	Ghana Food and Drug Authority: Guideline for Donation of Drugs	No date	1–10	https://rome.mfa.gov.gh/uploads/SectionImagWithLinks/3279_Guidelines-for-Donations-and-Voluntary-Medical-Outreach-Programmes-in-The-Health-Sector-of-Ghana.pdf
Lao People’s Democratic Republic	LDC recipient country	No	Regulation on Drug and Medical Products Donation	2003	All 12	http://www.fdd.gov.la/download/contentfiles/2017-07-21_101043am_Regulation%20on%20Drug%20Donation%20English%20version.pdf
Liberia	LDC recipient country	No	Liberia Medicines and Health Products Regulatory Authority (LMHRA): Regulations for Donation of Medicines and Health Products	2021	All 12	https://lmhra.gov.lr/regulations-on-donations-lmhra-05-01-22/
Myanmar	LDC recipient country	No	National Medicines Policy	2019	In development	http://www.doms.gov.mm/wp-content/uploads/2019/12/National-Medicines-Policy.pdf
Nepal	LDC recipient country	Yes	Guidelines for Drug Donations to Nepal	2021	All 12	https://pharmainfonepal.com/guidelines-for-drug-donation-to-nepal
Nigeria	Lower-middle income recipient	Yes	National Agency for Food and Drug Administration (NAFDAC) Guidelines for Donated Medical Products in Nigeria	2021	1–5, 7–10	https://www.nafdac.gov.ng/wp-content/uploads/Files/Resources/Guidelines/DR_And_R_Guidelines/Guideline-for-Donated-Medical-Products-in-Nigeria.pdf
Papua New Guinea	Lower-middle income recipient	Yes	Papua New Guinea Ministry of Health: The National Drug Policy for Papua New Guinea	1998	All 12	https://www.health.gov.pg/pdf/NATDRUGPOLICY.pdf
Philippines	Lower-middle income recipient	Yes	Guidelines in the Importation, Facilitation and Management of Foreign Donations involving Health and Health-Related Products	2020	1, 2, 4, 6, 7, 10, 12	https://www.fda.gov.ph/wp-content/uploads/2021/08/Administrative-NO.-2020-0001.pdf
Sierra Leone	LDC recipient country	No	Government of Sierra Leone Ministry of Health and Sanitation: Guidelines for the Donation of Medicines, Medical Supplies and Equipment to Sierra Leone	2004	All 12	https://www.humatem.org/telecharger_document_base_documentaire/106
Somalia	LDC recipient country	No	The Somali National Medicines Policy	2014	In development	https://moh.gov.so/en/wp-content/uploads/2020/07/Somalia-National-medicine-policy-2014.pdf
South Africa	Upper-middle income recipient	Yes	Medicine Control Council: Medicine Donations to South Africa	2010	All 12	https://www.sahpra.org.za/wp-content/uploads/2020/01/e39e6a7a5.08_Donations_May03_v2.pdf
South Sudan	LDC recipient country	No	Guidelines for the Donation of Medicines, Medical Supplies and Equipment for Southern Sudan	No date	All 12	https://www.humatem.org/telecharger_document_base_documentaire/109
Timor-Leste	LDC recipient country	No	National Drugs and Medicines Policy	2010	All 12	https://www.ms.gov.tl/sites/default/files/2021-09/TL_NDP_Drug_policy%20June%2024%202010%20to%20print%2028%20June%202010%20FINAL_0.pdf
Tuvalu	LDC recipient country	Yes	Pharmacy and Therapeutic Products Act	2016	All 12	https://tuvalu.tradeportal.org/media/PharmacyandTherapeuticProductsAct2016_1.pdf
Uganda	LDC recipient country	Yes	Procedure for Importing Drug Donations in Uganda	2018	1–6, 10	https://www.nda.or.ug/procedure-for-importing-drug-donations-in-uganda/
United Republic of Tanzania	LDC recipient country	Yes	Guidelines for Importation and Exportation of Pharmaceutical Products and Raw Materials	2011	1–10	https://trade.tanzania.go.tz/media/Guidelines%20for%20importation%20and%20exportation%20of%20pharmaceutical%20products.pdf
Zambia	LDC recipient country	No	Pharmaceutical Regulatory Authority (PRA): Guidelines for Drug Donations to the Republic of Zambia	2005	1–10, 12	https://www.mm3admin.co.za/documents/docmanager/2D5ED792-878 C-4371-9575-8281A96BBB26/00031197.pdf
Zimbabwe	Lower-middle income recipient	Yes	Guidelines for Medicines Donations to the Republic of Zimbabwe	2012	All 12	https://zdhr.uz.ac.zw/bitstream/handle/123456789/1371/Guidelines%20for%20Medicines%20Donations%20to%20Zim_Draft%201.pdf?sequence=1&isAllowed=y

*based on McDonald et al. [8]

**Table 2:** Breakdown of drug donation policies among actors who have donated medicines

Type of Actor	Total recorded participants in donation activities*	Number (%) with policies on donations
**Country donor**	5	1 (20)
**Pharmaceutical company donor**	20	3 (15)
**NGO donor**	15	3 (20)
**Country recipient**	26	13 (50)

*based on McDonald et al. [8]

### Challenges to implementing the 2010 WHO guidelines

Despite our extensive literature search we were only able to identify a small number of articles that specifically focused on difficulties that donors had in implementing donations and/or made recommendations for revisions to the *Guidelines.* Donation alignment with the 2010 WHO *Guidelines* lagged in five key respects. First, both donors and recipients have faced challenges in relation to the management of information about medicine donations. This is exemplified by reports of a lack of communication between stakeholders, poor operational oversight, a lack of pharmaceutical product record keeping, and irrelevant or unsolicited donations [10]. Second, recipients report problems with medication presentation. Donated products have contained pharmaceutical labels written in recipient countries’ non-official languages or with limited quality assurance documentation [11]. Third, some of the literature pointed out that there is a risk that large-scale donations may distort pricing in the global commercial market for pharmaceuticals [12]. Fourth, perceptions that all donations are good donations undermine WHO *Guidelines* principles enshrining respect for a recipient country’s specific medicine needs [2]. Finally, many authors report ongoing concerns about the sustainability of donations – either as short-term relief unable to solve structural medicine shortages or long-term policies that undermine recipients’ self-sufficiency [13]. Table 3 summarizes the barriers identified to implementing the WHO recommendations.

**Table 3:** Challenges to implementation of the WHO Guidelines

Area	Specific issues
Information management	• Transparent communication between stakeholders • Poor operational oversight • Lack of pharmaceutical product record keeping • Irrelevant and unsolicited donations
Medication presentation	• Labels of pharmaceuticals written in languages unfamiliar to recipient countries • Limited quality assurance
Influence from the pharmaceutical industry	• Large-scale donations could distort the global medicine commercial market
Belief that donations are inherently good	• Perception that there are benefits for recipients in all cases
Donation sustainability	• Short-term relief efforts are confined to providing assistance in a small window of time • Long-term solutions can encourage recipients to become too dependent on external handouts

## Discussion

We analyzed 38 policies on donations from donor countries (1 policy), pharmaceutical companies (6 policies), NGOs (6 policies) and recipient countries (25 policies). Twenty-five policies were implemented in 2010 or later and 16 of those incorporated all 12 recommendations from the most recent *Guidelines* revision. We also found that the large majority of donors in all three categories who had documented participation in donations either did not have policies governing those donations or those policies were not publicly available.

Recommendations 11 and 12 were the most likely ones to be missing, primarily in the recipient countries. These two recommendations focus on ensuring that the value of donated medicines match the wholesale price of the equivalent in the recipient country, and that all logistical donation costs are covered by the donors, respectively. The lack of implementation of these recommendations into recipient countries’ policies could allow for donors who also may have not incorporated them into their policies to leave wholesale price matching and donor accountability of logistical costs out of the conversation. This absence creates a cycle whereby recipient countries that do not have highly developed economies are sometimes be forced to pay out of pocket for some of the costs associated with the medicines they are being donated to them, creating a financial burden to them.

In addition, some authors have raised the risk that large-scale donations may distort pricing in the global commercial market for pharmaceuticals [13]. This distortion could affect alignment with the 2010 WHO *Guidelines* because recommendation 11 states that all donated medicines should match the wholesale price of the generic equivalent in the recipient nation. If this recommendation is not fulfilled, prices with higher mark-ups coming from donor countries can make the overall pharmaceutical market too expensive for citizens in recipient countries and pose another barrier to essential healthcare. Recommendation 12 also states that donors should cover logistical costs in the donation process, as recipient countries should not have to incur additional financial burdens if they are facing a crisis or do not have a large enough healthcare budget.

The findings from our study point out the need for stakeholders to focus on the economic implications that their donations can have on other participating actors, especially recipient countries. In-other words, donations that do not comply with the *Guidelines* can hurt those countries they are intending to help by creating additional financial burdens. This precaution and consideration when incorporating the WHO recommendations into donation policies can mean the difference between pharmaceutical aid that is genuinely beneficial, and aid with underlying financial hinderances for recipients to endure once the medicine arrives [14].

We also found that there are many actors across all categories (donor and recipient countries, NGOs and pharmaceutical companies alike) that do not currently have medicine donation policies or possibly their policies are not accessible to the general public for reference. This was evident in our search especially amongst donor countries, as we were only able to discover medicine donation policies for 1 out of the 12 countries.

A lack of transparency in medicine donation policies must be addressed. If actors do not currently make medicine donation policies publicly available, other actors and individuals outside of their organization will lack valuable information which may impact potential donation partners who are looking to work with them to donate or receive medicines. If all medicine donation actors shared their current policies, actors could hold each other accountable and provide suggestions to improve their policies to be more in line with the 2010 WHO *Guidelines*. Actors could also hope to gain more trust from the general public if they make audiences aware of how medicine donations operate within their organizations, which could in turn provide them with more support, funding opportunities and international partnerships in the future.

The difficulties in implementing the guidelines that we identified in the literature strongly suggest the need for a new round of revisions.

### Recommendations

There are six suggestions for revision to the *Guidelines* recommendations that were synthesized based on our findings. They are as follows:

1. Include a distinct definition of who is a “recipient” in the donation process to improve clarity between stakeholders. This will allow for all parties involved in the donation process to understand their roles and the roles of others to maximize efficiency and communication.
2. Synthesize a list of successful global case study examples to assist guideline users.
3. Provide a mechanism for recipient countries to decline donations.
4. Use flowcharts to represent the mechanics of medicine donations.
5. Create visuals to illustrate how stakeholders can efficiently operate pharmaceutical interventions to provide aid to recipients.
6. Donors that do not have a donation policy should reference the 2010 WHO *Guidelines* to lead their medical humanitarian aid efforts.

Overall, we also found very minimal academic and public discourse on emergency preparedness in the context of disaster relief efforts and medical donations. One potential area for further action, is the implementation of “virtual” donation exercises for disaster preparedness training [13]. This would enable users to better plan for receiving donations of essential medicines by simulating real life scenarios in different settings of urgency and could assist in shaping disaster relief protocols and procedures.

### Limitations

Although we believe that our search criteria were comprehensive, we may have missed finding policies of some donors and recipients, including because the policies were not publicly available. It is almost certain that there are other donors and recipients who fell outside our inclusion criteria who may have policies that we failed to capture and our results do not apply to these actors. We also did not evaluate how rigorously policies were applied by either donors or recipients. Finally, even in the absence of policies, donors in all three categories are making donations and countries are receiving donations.

## Conclusions

While the current WHO *Guidelines for Medicine Donations* provide a foundation for medicine donations, the evidence we found points to the need for further policy development to meet current challenges and more widespread adoption of the *Guidelines* by all actors. Understanding what has and has not worked in practice is essential for ensuring that the *Guidelines* remain up to date. Our findings suggest that both donors and recipients could further align their policies with the existing *Guidelines* and both groups should be consulted on any revisions to ensure that their experiences are reflected and their needs are addressed. Donors and recipients should also produce publicly available annual reports to summarize their donation efforts each year. Full implementation of the *Guidelines* would maximize the positive impacts of medicine donations for countries in need.

### Electronic supplementary material

Below is the link to the electronic supplementary material.

Supplementary Material 1

## Data Availability

All data generated or analysed during this study are included in this published article and its supplementary information files.

## List of abbreviations

LDC: least developed country
NGO: Non-governmental organization
UN: United Nations
WHO: World Health Organization
