# Supplementary material for: Medicine donations: a review of policies and practices
Source: Global Health. 2023 Sep 1;19:67. doi: 10.1186/s12992-023-00964-3 (PMC10474724; doi:10.1186/s12992-023-00964-3)
Supplement: Supplementary file 1 — Supplementary Material 1 [file 12992_2023_964_MOESM1_ESM.docx]

**Supplementary File 1: Donor and recipient actors without policies/guidelines on drug donations**

**Least developed countries recipients**

Angola

Bangladesh

Benin

Burkina Faso

Burundi

Cambodia

Central African Republic

Chad

Comoros

Cote d’Ivoire*

Democratic Republic of the Congo*

Djibouti

Eritrea

Guinea

Guinea-Bissau

Haiti*

Kiribati

Lesotho

Madagascar

Malawi*

Mali

Mauritania

Mozambique

Niger

Rwanda*

Senegal

Sudan

Togo

Vietnam*

Yemen*

**Lower-middle income country recipients**

Egypt*

Indonesia*

Pakistan*

Palestine*

Tajikistan*

**Upper-middle income country recipients**

Ecuador*

Georgia*

Guatemala*

Jordan*

Mexico*

**High-income country recipients**

Chile*

**Country donors**

Brazil*

Canada

China*

France

Germany

Italy

Japan*

Saudi Arabia

Turkey*

United Kingdom

United States

**Pharmaceutical companies**

Abbott*

Alcon*

Apotex*

Bayer*

Biogen*

Boehringer Ingelheim*

Bristol Myers Squibb

Eisai*

Eli Lilly*

GlaxoSmithKline*

Immune Therapeutics Inc.*

Lundbeck*

Johnson and Johnson*

Pfizer*

Sanofi*

Swedish Orphan Biovitrum AB*

Takeda*

Zentiva*

**Non-governmental organizations**

Action Against Hunger

ActionAid Internationl

Alertnet

American Medical Resources Foundation*

Americares

Association of Medical Doctors of Asia (AMDA)

Bill Clinton Foundation*

BRAC

CARE

Danish Refugee Council

Emergency Nutrition Network

Global Humanitarian Assistance

Global Links*

Granted Wish Foundation*

Health Link

HealthNet TPO

Health Partners International of Canada*

Heart to Heart International*

International Committee of the Red Cross

International Health Partners*

International Medical Corps

International Rescue Committee

Jack Brewer Foundationn*

MAP International

MATTER*

Norwegian Refugee Council

Oxfam

Plan International

Save the Children UK

SHARE*

SPHERE

Team Canada Healing Hands*

United States of America Committee on Refugees

World Medical Relief*

World Vision USA

*Mentioned as donor or recipient in McDonald S, Fabbri A, Parker L, Williams J, Bero L. Medical donations are not always free: an assessment of compliance of medicine and medical device donations with World Health Organization guidelines (2009-2017). International Health 2019;11:379-402. doi:10.1093/inthealth/ihz004
